# The Toxic Origins of Disease

**DOI:** 10.1371/journal.pbio.0050193

**Published:** 2007-06-26

**Authors:** Liza Gross

## Abstract

Researchers say that endocrine-disrupting chemicals can permanently damage the developing organism and may even promote obesity. But the chemical industry doesn't want you to believe them.

**Researchers say endocrine-disrupting chemicals can permanently harm the developing organism and may even promote obesity. But the chemical industry doesn't want you to believe them.**

Never in his wildest dreams had Fred vom Saal pictured himself studying urethral outlet obstruction. Nor, for that matter, had he ever thought much about the causes of obesity. For most of his 30-year career, vom Saal, a developmental biologist at the University of Missouri, studied the harmful consequences of tiny changes in natural hormone levels at critical periods during the development of the brain and reproductive tract. But he began to include synthetic chemicals in his investigations when he learned that pesticides and other environmental contaminants caused reproductive defects in wildlife much like those seen in lab animals exposed to abnormal estrogen levels.

**Figure:**
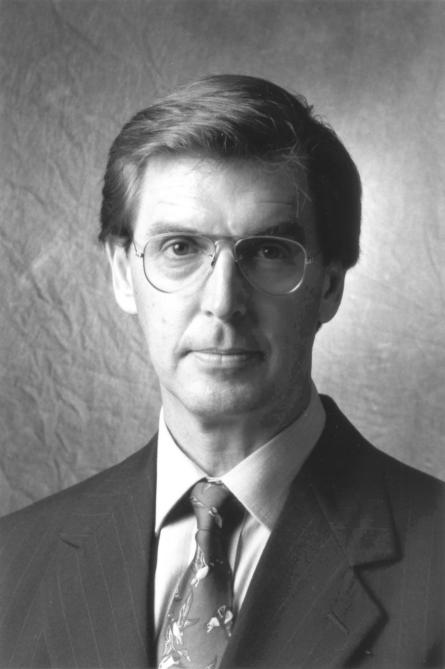
Fred vom Saal

During embryonic development, steroid hormones like estrogen control gene-expression programs to coordinate cell differentiation, growth, organogenesis, and metabolism. Adding extra estrogen—whether foreign (exogenous) or natural (endogenous)—can irreversibly alter these developmental processes by mimicking, blocking, or otherwise disrupting pathways that have been fine-tuned over millions of years to respond to minuscule changes in hormone levels. Diethylstilbestrol (DES) offered a tragic illustration of the risks of exposing a fetus to synthetic compounds that mimic the behavior of endogenous estrogen: the drug was prescribed to millions of pregnant women before doctors realized it was causing rare cancers in their daughters.

To understand how exogenous estrogens interfere with developmental pathways, vom Saal started by feeding pregnant mice minute doses of DES, along with the endogenous estrogen estradiol, which he had long studied. In both cases, giving the mother these estrogens when prostate development is occurring raised fetal estrogen levels ever so slightly, with profound consequences: male offspring experienced accelerated prostatic gland growth and showed permanent increases in both the number of androgen receptors (androgen mediates prostate differentiation) and the size of the prostate [1].

But it wasn't until vom Saal reported similar effects from a synthetic chemical still in mass production that his research focus, and his life, would take an unexpected turn. In 1997, vom Saal's group reported that feeding pregnant mice trace amounts of bisphenol A—the building block of polycarbonate plastics—caused enlarged prostates in male offspring, just as estradiol and DES had. “Our findings,” the researchers wrote, “show for the first time that fetal exposure to environmentally relevant parts-per-billion (ppb) doses of bisphenol A, in the range currently being consumed by people, can alter the adult reproductive system in mice”[2].

The next year, vom Saal's group showed that a similar treatment with bisphenol A also shrinks seminal vesicles, enlarges preputial glands (which produce sex pheromones), and reduces sperm efficiency [3]. The 1998 study, which observed these effects at a dose six times lower than a patient might swallow during application of a plastic dental sealant, immediately caught the attention of the chemical industry—and transformed Fred vom Saal into a tireless crusader against bisphenol A.

## An Obscure Chemical Enters the Limelight

For over 40 years, bisphenol A labored in relative obscurity as the feedstock for a wide range of commercial plastics and synthetic resins. Growing demand for polycarbonates—for products ranging from baby bottles to compact discs—drives the rapidly expanding multibillion-dollar market for bisphenol A, one of the highest-volume chemicals in commercial production [see related essay (published online 17 July 2007); doi:10.1371/journal.pbio.0050200]. Bisphenol A molecules, which are joined by unstable bonds to form polycarbonates and resins, leach from containers exposed to heat or highly acidic or basic compounds. Although bisphenol A's estrogenic activity was first reported in 1936, scientific interest in the chemical has recently increased along with evidence of its effects. And as the media increasingly cover these findings, the chemical industry has stepped up its attacks on those studying endocrine disruption.

“The moment we published something on bisphenol A, the chemical industry went out and hired a number of corporate laboratories to replicate our research. What was stunning about what they did,” vom Saal says with a mix of outrage and bemused disbelief, “was they hired people who had no idea how to do the work. Each of the members of these groups came to me and said, ‘We don't know how to do this, will you teach us?’”

Vom Saal videotaped his protocols for a group hired by Dow Chemical, and sent one of his students to England to teach AstraZeneca scientists the system. By 1999, a flurry of studies appeared from AstraZeneca [4–8], along with a collaborative effort sponsored by the Society of the Plastics Industry (SPI) from the labs of Dow, Shell, General Electric, and Bayer, the major bisphenol A producers [9]. (AstraZeneca does not make bisphenol A, but it produces a number of pest-control products that could face similar scrutiny.) None of the studies found that low doses of bisphenol A harm the developing prostate.

The next year, however, a study came out that supported vom Saal's findings [10]. Channda Gupta, then a professor of pharmacology at the University of Pittsburgh School of Medicine, fed pregnant mice low doses of bisphenol A, aroclor (an estrogenic pesticide), and DES, which she used as a positive control, as vom Saal had. (If animals fail to respond to DES, whose effects are well understood, it's a sign that the setup is flawed.) Gupta also observed increased prostate size and gland number in male offspring. When she placed the developing prostate in culture and treated it with the chemicals, she saw the same abnormal prostatic growth—indicating that the chemicals targeted the prostate directly.

Now vom Saal has gone even further with his rodent studies to show that bisphenol A activates androgen and estrogen receptor genes in the embryonic cells that give rise to prostate tissue [11]. This gene activity accelerates the proliferation of epithelial cells in prostate ducts, inducing prostate growth, and permanently increases the number of androgen receptors. “One of the consequences of that is that cells become hyper-responsive to hormones for the rest of the life of the individual,” he explains, “and that's a risk factor for humans for prostate cancer.” The overgrown prostate ducts also squeeze the urethra, constricting its passage out of the bladder—a condition similar to bladder outlet obstruction disease.

The year after Gupta's study appeared in *Experimental Biology and Medicine*, the journal ran a commentary, written by Barbara Elswick, Frederick Miller, and Frank Welsch of the Chemical Industry Institute of Toxicology (CIIT), faulting her analytical methods and conclusions [12]. The CIIT was “created by farsighted chemical industry leaders,” according to its Web site, and is funded by the American Chemistry Council (ACC). The CIIT scientists, who no longer work at the institute, could not be reached for comment.

Gupta's response defended her original conclusions and pointed out: “It is interesting to note that the studies that failed to find an effect of this chemical are funded by the chemical industries, whereas positive findings are reported by independent academic laboratories. What is also clear is that scientists who choose to study a chemical of commercial importance are subjected to intense scrutiny by the chemical industry and by the scientists funded by these industries” [13].

## Low-Dose Effects: A Manufactured Controversy?

Endocrinologists know that hormones normally stimulate their receptors at low concentrations, while high concentrations can inhibit these pathways by saturating receptors. But the notion that a substance can produce effects at low levels that disappear at higher levels fundamentally challenges traditional toxicological approaches. Faced with conflicting reports of harm from a chemical in mass circulation, the Environmental Protection Agency (EPA) asked the National Toxicology Program (NTP) to review the evidence on bisphenol A. If the panel decided the evidence was compelling, it would mean that the EPA's current risk-assessment methods, which assume “the dose makes the poison,” are outdated. It would also mean that a significant share of the chemical industry's portfolio of pesticides and industrial chemicals—many of which are suspected endocrine disruptors—would be subject to a new standard of risk assessment, with potentially substantial financial implications.

“The moment we published something on bisphenol A, the chemical industry went out and hired a number of corporate laboratories to replicate our research. What was stunning about what they did . . . was they hired people who had no idea how to do the work.”

The EPA estimates safe human exposures to a chemical based on animal studies that find the lowest harmful dose and the highest benign dose, then dividing one of the doses by a safety factor to account for variable human sensitivities and uncertainties in extrapolating to humans. Experiments from the 1980s, which found the lowest harmful dose at 50 milligrams of bisphenol A per kilogram body weight per day, were used to calculate the current safe dose of bisphenol A (50 micrograms bisphenol A per kilogram body weight per day). “It's just through hand waving that the regulatory community says if you divide that number by 1,000 that it's a safe human exposure dose,” vom Saal says. “The public is told it's a safe dose, but nobody ever tests that.”

In its initial review in 2001 [14], the NTP panel decided there was “credible evidence” that low doses of bisphenol A can cause effects on specific endpoints, but that the effects had not been “conclusively established as a general or reproducible finding.” This equivocal conclusion did not sit well with industry groups, so the American Plastics Council (APC) commissioned its own review from the Harvard Center for Risk Analysis (HCRA), which has received funding from all the major bisphenol A producers and their trade groups [see the Box].

HRCA Director Joel Schwartz acknowledges that taking money from industries with a direct financial interest in a study's outcome is problematic. “We're not averse to getting money from industry, but it used to be that the money predominantly came from industry, and I don't think that's a good idea,” he says. “So we're trying to diversify, and one of the things we're trying to avoid is getting the phone company to pay for a study of cell phones and the diesel company to pay for a study of diesel engines.”

The HCRA report, commissioned before Schwartz's tenure, concluded that “the weight of the evidence for low-dose effects is very weak” [15]. Industry groups hailed the report as a comprehensive review by independent experts and quickly disseminated its findings. Yet the “comprehensive” report reviewed just 19 of 47 studies available in April 2002, and when it was published more than two years later, three panelists asked not to be listed as authors.

In a 2005 commentary, vom Saal and Claude Hughes, a reproductive endocrinologist who had served on the HCRA panel, argued that the report was already obsolete when it came out [16]. By the end of 2004, they had identified 115 published studies on low doses of bisphenol A. They also found a troubling trend. Ninety percent of government studies found significant effects of bisphenol A at doses below the EPA's lowest adverse effect level, but not a single industry study found any effect. Many of the industry studies, they pointed out, either used a rat strain with very low sensitivity to estrogen or misinterpreted failure to find effects with positive controls. Vom Saal and Hughes urged the EPA to conduct a new risk assessment on bisphenol A.

Hughes was unaware of the HCRA's historic ties with industry when he was asked to serve on the panel—“It said Harvard, I said legit, and showed up”—but having worked in the pharmaceutical industry, Hughes, now chief medical officer and vice president of RTI International, is sensitive to charges of industry bias. “I've had to remind people that we also pointed out that government-sponsored studies on bisphenol A have tended to be mostly positive. Is that a bias? You want good results so you get your grant renewed, don't you?” Hughes thought that raising the question of bias for stakeholders on both sides would trigger “the type of comprehensive review I had hoped would occur a few years ago. . . which was not something our small committee in Boston could begin to pull off.”

The HCRA panel focused mostly on “whole-animal toxicological studies,” which look at different endpoints than the more mechanistic studies do, Hughes says. “That doesn't let you look at changes in gene expression, changes in epigenetic control of gene expression. It doesn't let you look at what, say, for a human population might be of concern.”

Vom Saal and Hughes's call for a new risk assessment triggered another APC-sponsored report on bisphenol A, this time from Gradient Corporation [see the Box]. In 2006, Gradient issued its “updated weight of the evidence evaluation,” which the authors called an extension of the HCRA report [17]. And like the HCRA report, the review often cited studies that lacked positive controls or used the insensitive rat strain to conclude that bisphenol A produces no adverse effect on any endpoint. In one case, the authors acknowledged a finding of decreased sperm counts, but argued that it was unclear whether such an effect could be considered adverse. In reviewing evidence of chromosomal abnormalities, the report excluded a much-publicized study by geneticist Patricia Hunt [18]—which showed that low levels of bisphenol A trigger substantial increases in improper chromosome segregation—on the grounds that the study didn't measure a developmental or reproductive endpoint. The authors did not explain how chromosomally abnormal oocytes might produce normal individuals.

## Stepping into a Quagmire

Hunt, now with the School of Molecular Biosciences at Washington State University, started studying bisphenol A when she ran an experiment one week with normal results and then ran it the next, “and the control numbers were just completely bonkers.” Sterilizing the animals' cages with high heat and accidentally washing them with a high pH soap damaged their polycarbonate cages and water bottles. Hunt found that bisphenol A leached from the water bottles and into their drinking water, and likely contaminated their food and penetrated their skin. Rather than pinpoint the exposure routes, however, she wanted to determine whether bisphenol A caused the anomaly. She did that by deliberately exposing her mice to bisphenol A.

After publishing her results, Hunt says, industry “paid people to read our paper and provide talking points, things they could use to say, ‘Well, we aren't really sure about this, and well, they didn't do that, and this is suspicious.’ It was such a learning experience for me because I had never had a piece of my work scrutinized in such detail, and I always thought my scientific peers were going to be the ones who were going to be most critical.” Hunt had been “peripherally aware” of the disputes between academics studying endocrine disruption and industry, “but you never knew whether these people were credible scientists or not, and then when you step your own foot into it and you watch, industry really did try to run damage control on our work.”

Many of the industry studies [on bisphenol A] either used a rat strain with very low sensitivity to estrogen or misinterpreted failure to find effects with a positive control.

At the time, Hunt was studying chromosomal alignment in eggs undergoing division before ovulation. “Instead of lining up normally as they should, they were just not lining up at all,” Hunt explains. “We happened to be doing another set of studies where we were looking at eggs that completed the division and counting the chromosomes to see if they were normal or abnormal, and suddenly that data changed too. So we had two completely separate studies where we were studying what we thought were the precursors of the chromosomally abnormal egg, which would give rise to, for example, Down syndrome, and both sets of data showed us this sudden spike of abnormalities in completely normal mice.”

Hunt decided to test bisphenol A's effects on the initial stages of egg development [19], thinking she wouldn't find any, since no one thought that estrogen played a role in mediating these very early steps of chromosomal interactions during oogenesis. “But of course, we were completely wrong,” Hunt says. Exposing pregnant mice to bisphenol A “had a very striking effect on the very early stages of egg development” in the female fetus. “And this causes some real problems when those females become adults,” she explains. “So our data right now say there are several different times that are susceptible during development and that exposures during these times can dramatically disrupt chromosomal behavior and lead to chromosomally abnormal eggs.”

**Skeptics for Hire**

Ever since Rachel Carson first warned that synthetic chemicals pose long-term health risks, the chemical industry has defended its products by attacking the credibility of scientists reporting ill effects. This strategy involves hiring consultants and commissioning reviews that dispute the findings or minimize potential human risks from the chemical under study.

Founded in 1989, the Harvard Center for Risk Analysis (HCRA) long specialized in minimizing human health risks from its benefactors' products, starting with the tobacco industry. Internal documents released through the 1998 Tobacco Master Settlement Agreement reveal how HCRA founder John Graham signaled industry that its business was his business. In a 1991 fund-raising letter seeking US$25,000, Graham told Philip Morris's vice president of government affairs of his need “to learn more about the risk-related challenges that you face.”

Graham got his US$25,000 after a company scientist recommended meeting Graham because “he's a key player in all this risk analysis stuff that's currently going on in the government.” (Philip Morris had to reissue the check through one of its subsidiaries after Graham learned that the Harvard School of Public Health prohibited taking tobacco industry money.) The next year, according to a report from Public Citizen, Graham wrote to a top White House policy counsel official questioning whether an EPA report on the health risks of second-hand smoke relied on good science. In widely reported public statements, including testimony before the US Senate Regulatory Affairs Committee, Graham has characterized the HCRA's work as promoting a more reasoned public response to health, safety, and environmental hazards.

Gradient Corporation, the environmental consulting firm that wrote the HCRA follow-up review on bisphenol A, also cut its teeth on the tobacco industry. In the early 1990s, Gradient received nearly US$700,000 from RJ Reynolds, according to internal documents released through the tobacco settlement, to estimate dose-response relationships “for the purpose of comparing the biological activity of the New Cigarette with the standard cigarette.” The two Gradient principals working on the project offered RJ Reynolds expertise in cigarette toxicology and inhalation toxicology.

Gradient's game, says Richard Clapp, professor of environmental health at Boston University's School of Public Health, is product defense. Its services include promoting industry positions in op-eds, providing expert testimony in court, legislative, and regulatory proceedings, and issuing scientific reports. “They wind up defending people who are worried about liability,” Clapp says, “though they would say they're there to make sure that there's sound science behind whatever regulatory steps or litigation happens in this country.”

Environmental and public health experts know about Gradient, Clapp says, “because we run up against them when they testify against regulating some air pollutant and we're testifying in favor of it, and you realize, oh my god, we're on opposite sides. This is exactly what happened to me when I testified in the Salem City Council hearings that they ought to be putting more controls on the polluting smokestacks from a power plant in Salem, Massachusetts, because I said it's releasing particulate matter and undoubtedly having an adverse impact on the health of the community. The Gradient guy got up and said, ‘You have no proof of that and these studies on which these models are estimating the effects on health, they're all based on faulty science.’”

Lorenz Rhomberg, the lead author of Gradient's follow-up to the HCRA bisphenol A report, wrote a 2006 op-ed in the *San Francisco Chronicle* opposing a proposed California ban (Assembly Bill 319) of children's products made of plastics containing bisphenol A. The bill, Rhomberg argued, relied on “unproven speculation” and a “scientifically unorthodox hypothesis that tiny exposures of bisphenol A … might harm health.” AB 319 died in committee.

Via email, Rhomberg contends that the low-dose hypothesis is unorthodox because “it does tend to contradict the conclusions from evaluation of the whole body of evidence, because no clear mechanism for special low-dose effects . . . has been elucidated, and because the idea that ultra-low doses can have a distinct set of responses from those engendered by high doses is indeed a speculation.”

As of November 2006, 151 of 178 published low-dose animal studies reported harm from low-dose bisphenol A exposures. All of the studies reporting harm received government funding. In a 2006 lawsuit contesting a San Francisco ordinance banning toys and other plastic children's products containing bisphenol A, Rhomberg filed court declarations on behalf of the chemical industry and toymakers and retailers. He argued that “studies reporting effects are contradicted by other, more numerous studies, including the most comprehensive studies, that report the lack of [bisphenol A] effects on the same outcomes at similar doses.” Though the San Francisco ordinance passed, the section banning toys and children's products made with bisphenol A was repealed.

Calling distinctions between industry- and government-funded studies a “red herring,” Rhomberg insists that even government-funded studies fail to support the low-dose toxicity of bisphenol A. “To find over 150 studies now showing that BPA causes detrimental reproductive developmental effects,” he writes, “one has to include any study that reported any effect at any dose while setting aside the fact that those same studies typically report no effects at other (higher or lower) doses for the same endpoint, and they usually find no effect for a series of other endpoints that were also examined.”

In the court declaration, Rhomberg also argued that there's nothing to suggest that children would be more susceptible than adults to bisphenol A. “I think the mainstream opinion is that whether fetuses are particularly sensitive to a chemical is a chemical-specific issue,” he explains, “and there are many cases where there is no such special sensitivity.”

The notion that children cannot metabolize chemicals as efficiently as adults and are uniquely vulnerable to environmental toxicants is, in fact, widely accepted. Based on these principles, the National Children's Study (NCS) was designed to follow 100,000 US children from early pregnancy through age 21 to understand environmental links to dramatic increases in a wide range of chronic childhood diseases, including neurodevelopmental disorders, obesity, asthma, male reproductive birth defects, and certain cancers. Bisphenol A is among the environmental pollutants the NCS plans to track.

These findings suggest that bisphenol A poses risks to an exposed mother's fertility, the safety of her conceptions, and even to her grandchildren. In 2005, the Centers for Disease Control and Prevention (CDC) found bisphenol A in the urine of 95% of people tested [20], but Hunt is especially concerned that bisphenol A has been found in human blood, amniotic fluid, and cord blood. “The levels we're seeing in humans are really in the range of the levels we're testing in the mouse, and this stuff in humans does a good job of walking across the placenta.” The fact that estrogen leaches from plastics “raises interesting questions about in vitro fertilization (IVF),” Hunt says. “There's a big problem with aneuploidy [abnormal chromosome number] in IVF, and we've always assumed that part of that comes from the individuals . . . and the stimulation protocols themselves.” There are so many factors to control in IVF, she says, “they haven't even gotten to the idea of endocrine disruptors.”

Hunt is counting on prospective epidemiological studies to understand bisphenol A's effects on humans, and points to DES as proof that well-designed rodent studies can predict human risks. “We ‘ran the experiment’ with DES in humans first and then we went back and did all the rodent studies, and we found that, wow, the rodent model really was a good model for all these exposed humans.”

“Of course, there are lots of differences between mice and humans,” Hunt allows. “Mice bear litters for one thing. But when it comes to growing eggs, and this whole process of meiosis, the similarities are so striking that it makes the mouse a really powerful model, because the process is highly, highly conserved.”

## The Problem with Plastics

Ana Soto and Carlos Sonnenschein have been worried about what might be leaching from plastics ever since an estrogenic contaminant derailed their experiments in 1989. They were studying how estrogens regulate cell proliferation when, unexpectedly, the cells proliferated in their serum-supplemented medium whether or not estrogen was present. Previously, adding serum without estrogen inhibited the cells' proliferation while adding estrogens countered this effect, indicating that proliferation is the dominant state of cells in multicellular organisms and could be controlled only by inhibitory factors. “This anomalous behavior had never happened to us,” says Soto, who studies breast cancer and mammary gland development at Tufts University. “We knew that the only thing that could produce that proliferation was estrogen, and we didn't add it.”

After tracing the contaminant to the plastic tubes holding the culture media components, Soto contacted the manufacturer, which sent more tubes to test. When Soto and Sonnenschein sent their results to the manufacturer, it attributed the contamination to a new plastic formulation but refused to divulge the ingredients. “So we purified it, and it turned out to be nonylphenol,” Soto says, “the first estrogen discovered in plastic.”

Soto and Sonnenschein used the same cell line to test several other chemicals for estrogenic activity and found that many of them, like nonylphenol, behaved like estrogen. “And then we decided to ask a question,” Soto says. “If you give a hormone to an adult and then you stop, the effects for the most part regress. . . . But if you give something during development, you will produce alterations that persist throughout life, because you are altering the formation of the organism; not just the function, but the building of the organism. So we decided to study bisphenol A and whether it would affect the female genital tract, the mammary gland, and the neuroendocrine system.”

“Now the industry will say that animals are not humans, which you can say as much as you wish, but that brings us to a situation; in order to know what is happening to humans, what are you going to do? Intoxicate pregnant women on purpose?”

Soto exposed pregnant rodents to “minuscule doses of bisphenol A, the same doses that humans are exposed to, according to the CDC.” In rats, this treatment produced overweight female offspring; in mice, adding the estrogenic chemical produced female offspring that behaved like males. Both rats and mice also had altered ovarian cycles. In a second round of experiments in mice, in utero bisphenol A exposures induced changes in mammary gland development that began in fetal life and persisted. “When they reach puberty and beyond, we observed that there is an increase in the structures where cancer arises, and there's an increased sensitivity to estrogen,” Soto says. She also saw increased mammary duct density, the rodent equivalent to human mammographic density—a risk factor for women.

The mouse isn't a good model for breast carcinogenesis, Soto explains, so she used rats to study mammary carcinogenesis. At environmental doses, in utero bisphenol A exposures “produced significant increases in the amount of intraductal hyperplasias,” or preneoplastic lesions. At higher doses, she observed carcinomas in situ. “This parallels the finding that women exposed in utero to DES are now showing more propensity to develop breast cancer of the estrogen-sensitive type.”

The changes in the breast and genital tract were expected, Soto says, but some of the behavioral effects and obesity came as a complete surprise. “We were looking at an estrogen thinking it was going to affect the reproductive system and mammary gland only, but then these two other things emerged without us ever imagining that.”

Bisphenol A might induce epigenetic changes by altering patterns of DNA methylation, a chemical modification that controls gene expression, or by activating or silencing genes at the moment of exposure during a critical period of development. Soto is pursuing these possibilities. “A single exposure during a point of vulnerability may suffice,” Soto says. “You know the thalidomide story. You can have thalidomide every day of your life and you will be fine. But [take it] at certain times during pregnancy, your child will end up with no arms.”

As far as Soto is concerned, bisphenol A threatens public health. “Now the industry will say that animals are not humans, which you can say as much as you wish, but that brings us to a situation; in order to know what is happening to humans, what are you going to do? Intoxicate pregnant women on purpose? In any case, we're already exposing people, because 95% of us have bisphenol A in our urine, so the experiment cannot even be done.”

## The Chemical Link to Obesity

For the most part, researchers investigating endocrine disruption had focused primarily on behavioral and reproductive consequences. But over the past few years, it's become clear that some of the synthetic chemicals that disrupt the endocrine system also induce weight gain [21]. What's more, production of these chemicals closely tracks the rise of obesity. Retha Newbold started studying the reproductive consequences of in utero DES exposure after physicians stopped prescribing the drug in 1971. She always noticed her animals were heavy, as Soto had, but didn't think much of it. “It's one of those things that if you've been working with your mice for 30 some years, you notice right off,” says Newbold, a developmental biologist at the National Institute of Environmental Health Sciences Environmental Toxicology Program. “I can tell you simply by the size of the animal which is DES-exposed and which isn't.”

Newbold's lab was among the first to publish animal studies showing adverse effects in male offspring exposed to DES in the womb. “At that point, clinicians started looking at the male offspring [of mothers who took DES], and they had very similar things to what we were reporting in the mouse model,” Newbold says, including retained testes and hypospadias. She also studied DES's effect on the female mouse reproductive tract, and found various genital lesions, malformations, and abnormalities, as well as reduced fertility—replicating the aberrations reported in DES daughters.

“I think everyone, including myself, always assumed that the reproductive tract was the target tissue. And now we're beginning to realize that it isn't the only target tissue,” Newbold says. “We found out that brain is a target, bone is a target . . . and now the new target is adipocytes.” During prenatal and neonatal development, adipocytes (fat cells) receive the instructions they need to function properly throughout life. “These conversations between cells, which are set up during prenatal development, that's what's being interrupted,” Newbold explains. “And that's why we see these long-term changes.”

Vom Saal discovered a link between estrogen and obesity when AstraZeneca scientists tried to replicate his results on bisphenol A and prostate development. “Now the chemical corporation people, who didn't know where the prostate was, did not replicate the results,” vom Saal wryly notes. “But they did something very interesting. They produced obese animals.”

AstraZeneca scientists found no differences in the prostates of animals fed bisphenol A, DES, or normal mouse chow. Yet in 25 years of using that mouse strain, vom Saal had never seen an animal as big as those recorded by the AstraZeneca group. And, it turned out, all the rodent groups had enlarged prostates after all—which could explain why no differences were reported. The AstraZeneca scientists had achieved with normal food what vom Saal had done with bisphenol A. But how?

It seemed plausible that estrogenic contaminants in the food had estrogenized the animals to the point where negative controls appeared treated and the other groups could no longer respond to DES and bisphenol A. Vom Saal tested different foods to understand how nutrients might influence estrogen pathways. When he removed all the soy-derived plant estrogens from the mother's diet, he was astonished to see endogenous estradiol levels in the fetus rise, and the offspring become “horrifically obese.”

Adding the weak plant estrogens back in the diet suppressed the far more potent endogenous estradiol, he discovered, by inhibiting an enzyme required to make it. Feeding pregnant mice chow without plant estrogens mimicked the effect of giving them bisphenol A by stimulating increased production of estradiol. Yet when vom Saal fed estrogen to adult mice, their fat pads disappeared. What these results demonstrate, vom Saal explains, is that sensitivity to estrogenic chemicals, and their potential to cause harm, is very life-stage specific. That's one reason he's worried about supplemental estrogens during pregnancy: estrogen actually causes embryonic cells to differentiate into fat cells, a process called adipogenesis.

Recent evidence suggests that a class of ubiquitous environmental pollutants called organotins can also stimulate adipogenesis and interfere with energy balance [22]. Bruce Blumberg, associate professor of developmental and cell biology at the University of California, Irvine, calls these compounds “obesogens.” Organotins like tributyltin, once routinely painted on ship hulls to discourage barnacle growth, contaminate the marine food chain, causing sex reversal and other reproductive abnormalities in marine animals.

Blumberg's group discovered that organotins can activate two nuclear hormone receptors—peroxisomal proliferator-activated receptor γ (PPARγ) and retinoid X receptor (RXR)—that co-regulate the expression of genes involved in lipid homeostasis and adipogenesis. Inappropriate activation of these receptors, Blumberg says, may promote long-term changes in fat cell behavior and number that favor obesity. “When animals are exposed prenatally to organotins, it reprograms their metabolism such that if they're never again exposed, they still gain weight,” Blumberg says. “Even with normal diet and normal exercise, they get significantly fatter than animals that are not exposed.” The same thing will happen with a variety of chemicals, he adds, not just organotins.

“The big question is why. Is it because we've made more fat cells? We have some evidence that suggests that's one thing that's happening. Are the cells more efficient at storing fat than they would be otherwise? Is the body predisposed to divert more calories into fat than would be otherwise, the so-called thrifty phenotype?”

In the thrifty phenotype hypothesis, undernutrition in the womb programs metabolic systems to expect a postnatal world of undernutrition. From an evolutionary perspective, genes that promote insulin resistance (thereby limiting glucose uptake) and fat storage would prove advantageous in times of famine. But in a world of fast foods, empty calories, and supersized meals, the same genes would promote obesity, insulin resistance, and type 2 diabetes. Interestingly, a class of drugs used to treat type 2 diabetes (called thiazolidinediones) activates PPAR to reverse insulin resistance in muscle and liver, but in doing so increases fat mass by facilitating triglyceride uptake in adipocytes.

When vom Saal generates growth-restricted mouse pups by exposing mothers to bisphenol A, the babies go through a “ballistic postnatal growth period.” A second group of mice starts out “really heavy” and stays that way. Vom Saal's two types of obese mice have 430 genes with different activity in their fat cells, exhibit substantial differences in glucose tolerance and leptin levels (leptin regulates appetite and energy expenditure), and lose weight at different rates.

Though understanding the underlying causes of these differences will take “multiple lifetimes of work,” vom Saal says, it's clear that both animals end up heavy in entirely different ways, with entirely different physiological, fat metabolism, and regulatory systems. “We think that environmental chemicals like bisphenol A are likely to target subpopulations of individuals that are rendered very sensitive to these chemicals by virtue of their genes, genetic background, maternal–fetal interactions . . . and the amount of hormones they're exposed to.”

The connection between fetal growth restriction, environmental estrogens, and obesity risk may be especially relevant for infertile couples, who are increasingly opting for IVF. For various reasons, many IVF babies are born premature and growth-restricted. Vom Saal worries that exposing this “highly sensitive subgroup” of babies to environmental chemicals that lead to accelerated postnatal growth will permanently alter their capacity to metabolize even normal diets and predispose them, like the mice in his experiments, to a lifetime of obesity.

Vom Saal acknowledges that trying to unravel all the “phenomenally complex” interactions and components that contribute to obesity is “like chipping away at the pyramid,” but he has no doubt that animal studies on bisphenol A's effects have relevance to humans. “This chemical is harming snails, insects, lobsters, fish, frogs, reptiles, birds, and rats,” vom Saal says, “and the chemical industry is telling people that because you're human, unless there's human data, you can feel completely safe.”

For information on products containing bisphenol A, go to http://www.bisphenolafree.org/ (click on the Smart Plastics Guide) and http://www.ewg.org/reports/bisphenola/execsumm.php.
